# Examining the Joint Effect of Multiple Risk Factors Using Exposure Risk Profiles: Lung Cancer in Nonsmokers

**DOI:** 10.1289/ehp.1002118

**Published:** 2010-10-04

**Authors:** Michail Papathomas, John Molitor, Sylvia Richardson, Elio Riboli, Paolo Vineis

**Affiliations:** 1 Department of Epidemiology and Biostatistics, Imperial College London, United Kingdom; 2 Department of Mathematics, Coventry University, Coventry, United Kingdom

**Keywords:** air pollutants, Bayesian inference, clustering, combined effect, gene/environment interactions

## Abstract

**Background:**

Profile regression is a Bayesian statistical approach designed for investigating the joint effect of multiple risk factors. It reduces dimensionality by using as its main unit of inference the exposure profiles of the subjects that is, the sequence of covariate values that correspond to each subject.

**Objectives:**

We applied profile regression to a case–control study of lung cancer in nonsmokers, nested within the European Prospective Investigation into Cancer and Nutrition (EPIC) cohort, to estimate the combined effect of environmental carcinogens and to explore possible gene–environment interactions.

**Methods:**

We tailored and extended the profile regression approach to the analysis of case–control studies, allowing for the analysis of ordinal data and the computation of posterior odds ratios. We compared and contrasted our results with those obtained using standard logistic regression and classification tree methods, including multifactor dimensionality reduction.

**Results:**

Profile regression strengthened previous observations in other study populations on the role of air pollutants, particularly particulate matter ≤ 10 μm in aerodynamic diameter (PM_10_), in lung cancer for nonsmokers. Covariates including living on a main road, exposure to PM_10_ and nitrogen dioxide, and carrying out manual work characterized high-risk subject profiles. Such combinations of risk factors were consistent with *a priori* expectations. In contrast, other methods gave less interpretable results.

**Conclusions:**

We conclude that profile regression is a powerful tool for identifying risk profiles that express the joint effect of etiologically relevant variables in multifactorial diseases.

It is well known that chronic diseases such as cancer arise from the combination of several risk factors, including environmental exposures and genetic susceptibilities. An analytical approach that does not investigate complex multifactor effects is likely to be ineffective in explaining the onset of most common diseases. However, the investigation of interactions with traditional statistical methods has limitations related to low power and model constraints.

We have extended profile regression (Molitor et al. 2010), a novel Bayesian approach for the study of the joint effect of several covariates, to the case–control study design and have applied it to the investigation of lung cancer risk factors in nonsmokers within the European Prospective Investigation into Cancer and Nutrition (EPIC). This multicenter European study was coordinated by the International Agency for Research on Cancer and Imperial College London. More than 520,000 healthy volunteers were recruited in ten European countries (for a detailed description of EPIC, see Bingham and Riboli 2004). We analyzed both environmental and lifestyle exposures (air pollutants, physical activity, anthropometry) and genetic susceptibility (candidate genes). All of these variables were expected to be weak risk factors individually. We restricted our analysis to never smokers and ex-smokers who quit smoking at least 10 years prior to enrollment. We removed current and recent smokers from the analysis because the large effect of smoking can potentially mask the effect of other important risk factors.

## Materials and Methods

### Subjects

GEN-AIR (molecular changes and genetic susceptibility in relation to air pollution and environmental tobacco smoke) is a case–control study nested within the EPIC cohort; its aim is to study the relationship between various types of cancer and air pollution or environmental tobacco smoke. All cancers were diagnosed after recruitment. Only nonsmokers or ex-smokers who had not smoked for more than 10 years before enrollment were included in GEN-AIR. This cutoff point has been set in order to control for the potential residual confounding effect of smoking in ex-smokers. Three controls were matched per case. Matching criteria were sex, age (± 5 years), smoking status (never or former smoker), country of recruitment, and follow-up time. The number of subjects that met the protocol criteria was 4,051 (1,074 cases and 2,977 controls). The mean follow-up time for those GEN-AIR subjects was 89 months, with a minimum of 51 months and a maximum at 123 months (Matullo et al. 2006). The present study is on lung cancer only in nonsmokers (271 cases and 2,977 controls). Biological samples were obtained and DNA extracted to investigate whether lung cancer is associated with candidate single-nucleotide polymorphisms (SNPs) or with DNA adducts, which are markers of DNA damage. We had DNA available for 116 cases and 1,076 controls (the center in Malmo did not provide DNA; see Peluso et al. 2005). Matching on time since biological sample was collected (± 6 months, two controls per case) was introduced to avoid bias due to differences in sample degradation between cases and controls. We have complied with all applicable requirements of international regulations (including institutional review board approval). Human participants gave informed consent before the study.

### Environmental factors

As environmental factors of interest, we chose to include in our analysis three exposure variables related to air pollution, two characteristics of physical activity, and one anthropometric variable. We did not include information on environmental tobacco smoke because it was missing for a large number of participants and would have reduced the sample size available for profile regression analysis from 829 to 665 observations.

The association between air pollution and lung cancer has been studied in previous cohorts (Hoek et al. 2002) and in EPIC (Vineis and Husgafvel-Pursiainen 2005). We included in our analysis three measures of exposure to air pollution: residence on a main road (yes or no; detailed Internet maps were used to define if a home was located in a major street with validation using traffic count data from municipalities, local environmental agencies, or the Internet); exposure to ambient particulate matter (PM) air pollution [≤ 10 μm in aerodynamic diameter (PM_10_); < 30, 30–40, > 40–50, and > 50 μg/m^3^]; and exposure to nitrogen dioxide (NO_2_; < 30, 30–40, and > 40 μg/m^3^). Air pollution was assessed using routine measurements from air quality monitoring stations. Exposure was measured taking into account residence address at the time of enrollment and the average concentration of pollutants from the nearest monitoring stations (for details on the collection of the air pollution data, see Vineis et al. 2006).

The relationship between physical activity and various types of cancer has also been examined in the EPIC cohort, although results for lung cancer were not always consistent with previous studies in other populations (Steindorf et al. 2006). We evaluated two aspects of physical activity: physical activity at the work place (sedentary, standing occupation, manual work, or heavy manual work, with observations from 67 unemployed subjects of the 4,051 participants in GEN-AIR classified as missing), and a variable representing combined recreational and household physical activity (low, medium, or high based on the computation of sex- and center-specific tertiles that were used to rank the subjects within a center). In each EPIC center, professional and nonprofessional physical activity was assessed at baseline as part of a standardized questionnaire. In addition, we evaluated body mass index (BMI) using standard classifications (< 18.5, 18.5–25, and > 25 kg/m^2^). Almost all EPIC centers measured anthropometric factors at enrollment. The centers in France used self-reported baseline measurements of height and weight. The self-reported baseline measurements of the volunteers at the Oxford, United Kingdom, center were corrected for possible reporting bias (for more details on the collection of physical activity and anthropometric measurements, see Berrington de Gonzales et al. 2006).

### Genetic variables

DNA was extracted from 200–300 μL buffy coat in the Genova and Florence, Italy, laboratories. DNA was isolated and purified as described by Peluso et al. (2000). We chose to investigate two genetic markers that have been associated with lung cancer by other investigators (Matullo et al. 2006; Vineis et al. 2007): deletion polymorphisms in *GSTM1* (glutathione *S*-transferase mu 1 gene), and SNP 26304 (rs1799782 Arg194Trp) in *XRCC1* (X-ray repair complementing defective repair in Chinese hamster cells 1 gene). Genotyping was performed at the University of Aarhus, Denmark (*GSTM1*) and at the Institute for Scientific Interchange Foundation in Torino, Italy (*XRCC1* 26304).

In previous publications, the common deletion polymorphism in *GSTM1* has been associated with the presence of lung cancer (Carlsten et al. 2008; Malats et al. 2000). The *XRCC1* 26304 marker is a polymorphism in the *XRCC1* DNA repair gene. A protective effect against lung cancer was suggested by Matullo et al. (2006). Bulky DNA adducts are biomarkers of exposure to aromatic compounds and of the ability of the subject to metabolically activate carcinogens (resulting in adduct formation) and to repair DNA damage (resulting in adduct elimination) (Veglia et al. 2008). It is as yet uncertain whether DNA adducts predict the development of lung cancer. Studies in animals have demonstrated a role of DNA adducts in the development of tumors (Bartsch 2000). We measured bulky DNA adducts using relative adduct labeling (Gupta 1985).

### Statistical methods

In epidemiological studies, even with a moderate number of covariates, it is typically difficult to examine all possible interactions with standard regression techniques, because estimating a large number of parameters is required, and model selection quickly becomes cumbersome. Furthermore, risk factors are often correlated, which results in collinearity problems. Dimension reduction techniques have focused, broadly speaking, on deriving good prediction using a large set of covariates, or on clustering approaches. The first approach includes penalized methods such as the lasso technique (Tibshirani 1996) that select a set of predictors by shrinking the estimated effects of some covariates to zero. These methods allow the estimation of the selected regression coefficients but cause some bias. The second class of methods includes profile regression, which partitions observations into clusters that are relatively coherent with respect to exposure among observations within clusters and dissimilar with respect to exposure between clusters (Molitor et al. 2010). The link between the clusters and the outcome is characterized by an association parameter. Moreover, profile regression is framed in a statistical model-based paradigm that allows the computation of multiple estimates of association, including odds ratios (ORs) for the outcome for a particular profile relative to a baseline (reference) group, and the difference in the risk of the outcome between two specifically defined covariate combinations, along with appropriate evaluation of uncertainty. In this article, we report a comprehensive comparison of profile regression with logistic regression methods as well as with two non–model-based clustering methods, classification and regression tree (CART) and multifactor dimensionality reduction [(MDR) 2010)], described in detail below.

#### Profile regression analysis

Profile regression (Molitor et al. 2010) is a statistical approach designed specifically for the investigation of the joint effect of a moderate to large number of covariates. In profile regression, inference is based on how the covariate profiles of subjects are clustered into subpopulations, revealing important covariate patterns. The covariate profile of a subject becomes the basic unit of inference and is associated with a risk parameter that will be estimated. Clustering has been used before for the analysis of correlated data; see, for instance, Desantis et al. (2008) and Patterson et al. (2002) where Latent Class Analysis was employed. However, profile regression combines many recent statistical developments in a novel way, offering a number of advantages. First, as a Bayesian procedure, it allows the investigator to properly account for the uncertainty associated with the clustering of the subjects. Also, the number of clusters is random and not set in advance and is informed by the structure of the data (Ishwaran and James 2001). Finally, the outcome of interest influences cluster membership so that disease status and covariate patterns inform each other.

Our approach consists of an assignment submodel and a disease submodel, fitted together as a unit. The assignment submodel evaluates the probability that an individual is assigned to a particular cluster. We focus on categorical and ordinal covariates with *M_p_* categories for the *p*th covariate, and denote, for individual *i*, a covariate profile as ***x****_i_* = (*x*_1_, …, *x_p_*). Profiles are clustered into groups, and an allocation variable, *z_i_* = *k*, indicates the *k*th cluster to which individual *i* belongs. Let φ*_k_^p^* (*x*) denote the probability that the *p*th covariate in cluster *k* is equal to *x*. For each cluster, *k*, the parameters φ*_k_^p^* (*x*), *p* = 1, …, *P* define the prototypical profile for that cluster. For example, a parameter φ_1_*^pm^*^10^ (> 50 μg/m^3^) = 0.8 means that in cluster 1, the probability that a subject is exposed to > 50 μg/m^3^ is 0.8. If 0.8 is significantly higher than the average probability for this exposure category in the whole sample, then we can interpret group 1 as characterized by subjects who are, on the whole, exposed to a relatively high level of PM_10_.

To implement the assignment model with ordinal categories, we generalized the model described by Molitor et al. (2010) by introducing ordered threshold parameters and by linking the probabilities of the ordinal categories to the cumulative distribution of an underlying standard normal distribution as in Albert and Chib (1993). This specification does not force any particular relation between categories, other than the order of them [for more details, see Supplemental Material, Section S1 (doi:10.1289/ehp.1002118)].

To quantify the association between exposure groups and presence of disease, we assign to the *k*th cluster a parameter that measures its association with the outcome *y_i_* for individuals in cluster *k*; we call this the disease submodel. We denote this parameter as **θ***_k_*, so that *P*(*y_i_* = 1) = **θ***_k_* and is common to all individuals *i* in group *k*. For prospective cohort studies, **θ***_k_* is simply the risk of disease associated with the profile of exposure in group *k*. For case–control studies, **θ***_k_* is interpreted as an association parameter that quantifies the relation between exposure in group *k* and presence of disease. To adjust for confounders, we adopt a logistic regression formulation for the disease model logit [*P*(*y_i_* = 1)] = **β*w****_i_* + **θ***_k_*, where **β** denotes the coefficients for the confounder values that correspond to subject *i*. When the logistic formulation is adopted, the interpretation of the risk parameters **θ***_k_* changes. The risk parameters are interpreted as the baseline log odds of disease for an individual in group *k*, which is the log odds obtained when all confounders are set to their reference value of zero (see Molitor et al. 2010). Both clustering and disease models are fit in a unified manner via Bayesian inference techniques. Following standard practice, we have used the prospective likelihood in our modeling rather than a retrospective likelihood. This choice is not expected to have any adverse effect on our inferences because we consider categorical and ordinal risk factors and the relevant prior distributions are noninformative (see Seaman and Richardson 2004 and references therein).

The parameters of the model are estimated using Markov chain Monte Carlo (MCMC) methods (Gilks et al. 1996). Note that during the MCMC algorithm, we allow the number of possible groups to vary and adapt to the structure of the data.

After running the algorithm, we then derive, for ease of interpretation, an optimal number of groups together with an associated “best” partition of all the individuals into these groups. The optimal number of clusters is chosen with reference to a similarity matrix constructed in such a way that each (*i*, *j*) cell indicates the percentage of times two individuals appear in the same cluster throughout the run of the sampler. This is an estimate of the probability that individuals (*i*, *j*) belong to the same cluster and is invariant to changes in cluster labels or the number of clusters, which may vary between MCMC iterations. We then use a deterministic algorithm to find the clustering that most closely matches this similarity matrix [see Supplemental Material, Section S1 (doi:10.1289/ehp.1002118), or Molitor et al. 2010].

Our postprocessing approach uses the rich output of the MCMC sampler to assess parameter uncertainty corresponding to the subgroups that represent the best clustering. For example, suppose we are interested in the cancer risk associated with subgroup 1 in the best clustering, and this subgroup contains individuals 1, 3, and 5. We then simply calculate, at each iteration of the sampler, the average risk for these individuals. For example, 1,000 iterations will generate 1,000 average risks that can be used to derive this subgroup’s posterior distribution for cancer risk. If the algorithm generally puts individuals 1, 3, and 5 in the same cluster, this posterior distribution will tend to be relatively narrow, indicating a relatively high degree of certainty. Conversely, if the algorithm usually puts these individuals in disparate clusters, then the best clustering is less typical and the posterior distribution will tend to be relatively wide, which indicates greater uncertainty.

We extended the algorithm to deal with ordinal data using custom made MATLAB code and implementing the sampling procedure suggested by Cowles (1996) [see Supplemental Material, Section S1 (doi:10.1289/ehp.1002118)]. [The relevant code for the analysis in this article is available from the first author on request, it may also be downloaded from the Bayesian Gene eXpression web site (BGX 2010).]

We consider two relative measures to better interpret the association of exposure profiles with disease status. First, we consider the posterior distribution of (**θ***_k_*−**θ̄**), where **θ̄** is the average risk associated with the whole case–control sample in the study. From this posterior distribution, we report the probability *P*(**θ***_k_* > **θ̄**) or *P*(**θ***_k_* *<* **θ̄**), depending on whether **θ***_k_*, the risk associated with cluster *k*, is above or below the average risk associated with the whole population. A probability *P*(**θ***_k_* > **θ̄**) close to 1 (e.g., > 0.9) is interpreted as strong evidence that the *k*th group is associated with higher risk of disease than the overall study population. Similarly, a probability *P*(**θ***_k_* < **θ̄**) close to 1 suggests a relatively low-risk group. Second, to be consistent with the standard paradigm in case–control studies, we extend the computational setup described by Molitor et al. (2010) and derive the posterior mean of the odds ratio OR(**θ***_k_*, **θ**_Re_) = [**θ***_k_* (1 − **θ**_Re_)]/[**θ**_Re_ (1 − **θ***_k_*)], comparing the estimated odds of disease for the *k*th group and the odds for a baseline reference group (typically set to be the group with the lowest risk) together with the corresponding 95% credible interval for the OR.

#### MDR and classification tree analysis

MDR (Ritchie et al. 2001) is a model-free exploratory method for the investigation of interrelated risk factors. It was developed for the detection of gene–gene and gene– environment interactions (see, e.g., Cho et al. 2004). For interactions of order 2, MDR will examine all possible two-factor combinations among *P* covariates, (*x_p_*_1_, *x_p_*_2_), 1 ≤ *p*1, *p*2 ≤ *P*. For these two factors, each combination cell is characterized as high or low risk, depending on whether the ratio of cases to controls exceeds a certain threshold with a default value set to 1 (Hans et al. 2003). This is done using 90% of the data. Then, predictions are made for the remaining 10% of the data. The proportion of subjects for which a correct prediction is made is an estimator of the prediction accuracy. In fact, to minimize the variance associated with the estimates of the prediction accuracy, the data are divided in 10 equal parts and the procedure is repeated 10 times, eventually making predictions for the full data set. This 10-fold cross-validation procedure concludes with averaging the 10 prediction errors. The two selected factors are those that maximize the prediction accuracy among all pairs of factors. A similar procedure is applied to *p*, 3 ≤ *p* ≤ *P*, covariates. For the selected *p* covariates that maximize prediction accuracy, MDR gives two important scores: the mean prediction accuracy and the cross-validation consistency. The latter is the number of times a specific *p*-factor combination was identified as best in the 10 testing sets. Of all selected “best” sets of factors, containing from 2 to *P* covariates, the combination of choice should be characterized by high prediction accuracy and cross-validation consistency.

MDR is a special case of the classification and regression tree (CART) model (Bastone et al. 2004), where subjects can be classified into multiple groups (terminal nodes). We also implemented this more general approach, as available in the “tree” package of the R statistics software (R Development Core Team 2006), using the standard recursive partitioning model (Foulkes et al. 2004) to build a classification tree. This approach typically consists of a building step, where the splits that form the tree are decided, and a pruning step, where the size of the tree is reduced to achieve model parsimony. For the building step we use the “gini” criterion, so that the best split of the data in two groups is defined as the one that results in the greatest reduction of impurity, a quantity that describes the heterogeneity of a group of subjects with respect to the outcome (Bastone et al. 2004). For assessing the validity of splits during pruning we use 5-fold cross-validation, as described by Bastone et al. (2004), and choose the tree that minimizes the misclassification error. This is done by dividing the sample in five parts and then averaging the misclassification error across the five sets where predictions on the outcome are made. This gives an unbiased estimate of the misclassification error that corresponds to a tree of certain size.

#### Stepwise logistic regression analysis

Logistic regression is the most commonly used method for analyzing case–control studies. In our analysis, each of the risk factors is first introduced separately in a simple univariate logistic regression analysis, to individually assess how well each predicts the outcome. Next, the main inference proceeds by including all potential risk factors in a multivariate logistic regression model and using stepwise forward and backward selection. Forward selection starts with a model that only contains an intercept. Then, at each step, the algorithm adds to the model the covariate with the highest significance, given that this covariate is deemed to be significant in the presence of previously included covariates. Backward selection starts with the full model that includes all covariates. Then, at each step, the algorithm removes the covariate with the lowest significance, given that this covariate is not significant in the presence of the other covariates. For all logistic regression analyses we use the SPSS statistical software (version 17.0; IBM Corporation, Somers, NY, USA). We used the default SPSS significance levels: α = 0.05 for inclusion and α = 0.1 for removal.

#### Model fit

To check how well the different models fit the data, we used logistic-regression–type residuals. For a data set of size *n*, the quantity we used for our comparisons was


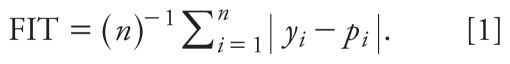


[For more details see Supplemental Material, section S2 (doi:10.1289/ehp.1002118).]

## Results

### Analyzed data and missing observations

The present study is on lung cancer only. This reduced the number of GEN-AIR subjects from 4,051 (1,074 cases and 2,977 controls) to 3,248 (271 cases and 2,977 controls). We performed the laboratory analyses in a subset of cases and controls. The *GSTM1* and *XRCC1* candidate SNPs were analyzed among 114 and 116 cases, respectively, and among 1,064 and 1,076 controls. We generated information on bulky DNA adducts for 115 cases and 1,072 controls. In contrast with previously published reports for GEN-AIR (Vineis et al. 2006, 2007), our analysis explored jointly genetic and environmental covariates. It was therefore important that a large proportion of subjects in the analyzed sample had fully observed genetic and environmental profiles. This is why we only used the intersection of genetic data and environmental data on traffic-related pollutants. For the profile regression analysis, we included 829 nonsmoking subjects (83 lung cancer cases and 746 controls) with full information on genetic variants, physical activity, BMI, and residential proximity to a main road. PM_10_ measurements were not available for 284 of these subjects (20 cases and 264 controls), including 49 (8 cases and 41 controls) that also lacked information on exposure to NO_2_. However, following the usual Bayesian paradigm, we stochastically imputed missing PM_10_ and NO_2_ data during the Gibbs sampling stage, as part of the simulation of the joint posterior distribution of all unknown quantities. This allowed us to include participants with incomplete environmental data while accounting for the uncertainty generated by the imputation of their exposure data.

Other statistical methods we employed (multivariate logistic regression using stepwise selection, MDR, CART) require data sets with no missing observations. Because of the missing PM_10_ observations, we based analyses with these methods on a reduced data set of 545 subjects (63 lung cancer cases and 482 controls) with complete PM_10_ data. When we considered one covariate at a time with simple univariate logistic regression, we had 545 subjects for investigating exposure to PM_10_, 780 subjects for investigating exposure to NO_2_, and 829 subjects for all other covariates; see Table 1 for a list of all risk factors included in our analysis.

### Profile regression analysis

**Figure 1:**
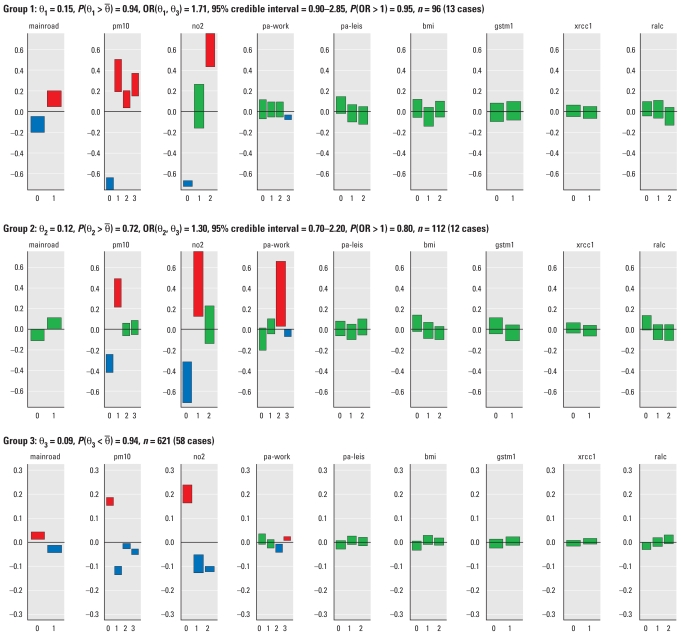
Profile regression output: 829 subjects analyzed with average risk. Abbreviations: bmi, BMI; gstm1, *GSTM1* gene; mainroad, residential proximity to a main road; no2, exposure to NO_2_; pa-leis, physical activity at leisure; pa-work, physical activity at work; pm10, exposure to PM_10_; ralc, bulky DNA adducts; xrcc1, *XRCC1* gene. For each covariate and each category, we provide the 95% credible interval for the difference between the probability φ*_K_^p^*(*x*) of attribute *x* in group *k*, and the corresponding average probability 
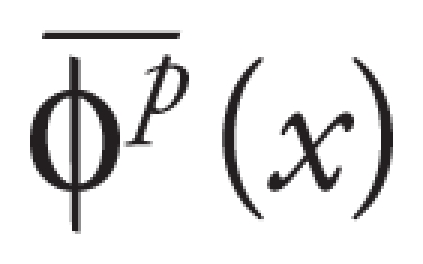
 in the whole population. Credible intervals are presented as bars. Green indicates that zero is contained in the 95% credible interval; red (blue) indicates positive (negative) credible intervals that exclude zero.

Profile regression revealed a typical grouping for the 829 subjects that consisted of three main subpopulations, with posterior means for the **θ***_k_* parameters varying from **θ**_3_ = 0.09 in group 3 to **θ**_1_ = 0.15 in group 1 (Figure 1). Because there was no predetermined baseline group in our analysis, we contrasted the odds of groups 1 and 2 with the odds of group 3, the group with the lowest **θ***_k_*. For group 1, which comprised 96 subjects, the probability that **θ**_1_ was higher than the average risk in the sample was 0.94. The OR comparing group 1 with group 3 [OR(**θ**_1_, **θ**_3_)] was 1.71, and the probability that this OR was > 1 was 0.95, giving a clear indication that group 1 was a high-risk group. Group 2 (112 subjects) was associated with moderate risk, with a probability of 0.72 that **θ**_2_ was higher than the average risk in the sample, and OR(**θ**_2_, **θ**_3_) = 1.3, *P*[OR(**θ**_2_, **θ**_3_) > 1] = 0.8. Group 3 contained a large number of subjects (621 out of 829) and was associated with a low risk of lung cancer relative to the population as a whole, with P(**θ**_3_ *<* **θ̄**) = 0.94. In the analysis presented here, we did not adjust for any additional covariates. Adjustment for the five matching variables [sex, age ± 5 years, smoking status (never or former), country (Greece, Italy, Spain, France vs. Germany, Netherlands, England, Sweden, Denmark, Norway), and follow-up time (end of follow-up is defined as date of censoring, death, emigration, or diagnosis, whichever came first)], using the logistic regression formulation of the disease submodel produced comparable results [see Supplemental Material, Table 1 (doi:10.1289/ehp.1002118)].

Figure 1 provides a graphical representation of the covariate profile associated with each group, including 95% credible intervals for the difference between the probability φ*_k_^p^* (*x*) of each value *x* of covariate *p* in group *k* and the corresponding average probability 
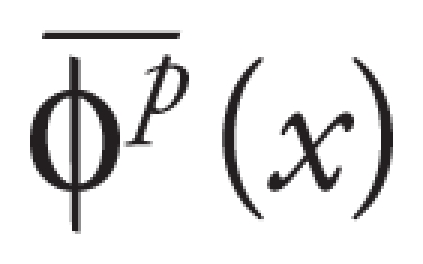
 in the whole population. A positive difference means that the characteristic was more common in this subgroup than in the population as a whole, and a negative difference means that the characteristic was less common. For example, the 95% credible interval for high exposure to NO_2_ in group 1 is > 0 (0.44, 0.86), indicating that this group was characterized by subjects who are more likely to have high exposure to NO_2_ than the overall population. Overall, group 1 included subjects who, on average, are more likely to live on a main road and be exposed to PM_10_ > 30 μg/m^3^ and NO_2_ > 40 μg/m^3^ than is the population as a whole but who are comparable to the rest of the study population with regard to physical activity, BMI, *GSTM1* and *XRCC1* classification, and bulky DNA adducts. Group 2 was characterized by subjects less likely than the overall population to be exposed to the lowest levels of PM_10_ and NO_2_ but more likely to be exposed to 30–40 μg/m^3^ PM_10_ and 30–40 μg/m^3^ NO_2_, and who were comparable to the overall population with regard to higher levels of PM_10_ and NO_2_ exposure. Other covariates in this group were comparable to the overall population, except that it included a relatively high proportion of manual workers. The low-risk group 3 was characterized by subjects who were less likely to live near a main road and more likely to have lower PM_10_ and NO_2_ exposures than was the overall study population. [See Supplemental Material, Figure 1 (doi:10.1289/ehp.1002118), for a visual two-dimensional representation of the clustering presented in Figure 1.]

In general, the two genetic factors and DNA adducts did not appear to contribute to the formation of the groups. When we compare the genetic profile of subjects in groups 1 and 2, we see that the lower risk group 2 contains a slightly higher than average proportion of subjects with the protective *GSTM1* marker and a slightly higher than average proportion of subjects with nondetectable DNA adducts. Increased DNA adduct levels in never-smokers have been associated in the literature with a variant in the *XRCC1* DNA repair gene (Matullo et al. 2001), but this effect is not clearly present in our analysis. Finally, increased levels of DNA adducts (above median) do not seem to have an adverse effect for subjects in group 3 with a low-risk environmental profile.

We also used profile regression to evaluate a reduced data set that we restricted to observations with complete PM_10_ data (545 subjects: 63 cases and 482 controls), so that we analyzed the same observations as for the other methods without imputation of the missing PM_10_ data. Results of this analysis were virtually identical to those based on the larger data set [see Supplemental Material, Table 2 (doi:10.1289/ehp.1002118)].

### MDR and classification tree analysis

**Figure 2:**
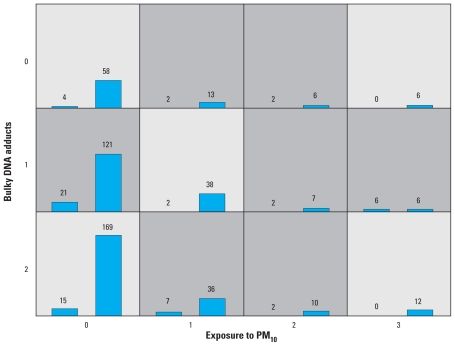
MDR graphical representation of how PM_10_ and relative adduct labeling combine to affect risk, derived with the standard MDR open-source software. Darker shading indicates combinations where the ratio of controls to cases is higher than 0.1156, the average control:case ratio in the sample.

Analysis using MDR indicates that a model including PM_10_ and DNA adducts is more predictive of the outcome than all other alternatives (Table 2). However, the graphical representation of how these two factors combine to influence the risk of lung cancer does not allow for a clear interpretation (Figure 2). Also, the low cross-validation consistency for this combination (6 of 10) indicates considerable uncertainty in choosing the overall best combination.

**Figure 3:**
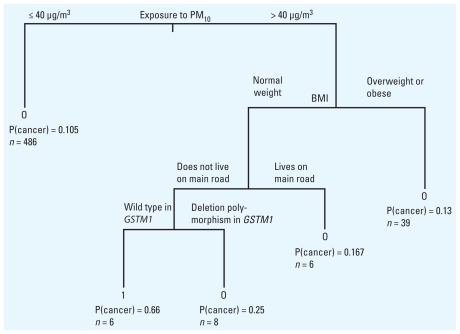
Classification tree using the “gini” impurity criterion. Pruning is done with 5-fold cross-validation. The average risk in the sample is 0.1156. *n* denotes the number of subjects corresponding to a terminal node. Values for P(cancer) indicate the average probability that a member of the subgroup will be a case.

Figure 3 presents the output from CART. The covariates that form the tree are PM_10_, BMI, living on a main road, and *GSTM1*. The other factors were deemed to be insignificant in reducing misclassification error and were removed during the pruning procedure described above (see “Statistical methods”). The results suggest that participants with PM_10_ exposures > 40 μg/m^3^ were at greater risk than those with PM_10_ < 40 μg/m^3^, which is consistent with the profile regression analysis. However, among subjects with PM_10_ exposures > 40 μg/m^3^, those living on a main road appeared to have a lower risk of lung cancer than those who did not live on a main road. Note the small number of observations in this node (we compared 14 subjects who lived on a main road with 6 who did not live on a main road) and the lack of any uncertainty evaluation. In addition, NO_2_ was not selected as a predictor in the formation of the tree. These later results are not consistent with *a priori* expectations.

### Logistic regression analysis

Individually, *p*-values ranged between 0.094 for physical activity at work and 0.914 for *XRCC1* [see Supplemental Material, Table 3 (doi:10.1289/ehp.1002118)], whereas no risk factor was selected in a multivariate logistic regression with forward or backward selection as model choice tool. Finally, no two-way interactions were significant, again using forward or backward selection.

### Model fit

A small exercise on model fit, presented in the Supplemental Material, section S2 (doi:10.1289/ehp.1002118), indicates that profile regression’s ability to reveal apparent complex effects does not come at a cost for goodness of fit.

## Discussion

Profile regression has been developed to analyze complex data sets in which multiple risk factors are likely to interact in the etiology of common multifactorial diseases. We extended profile regression to the case– control study design, allowing for the analysis of ordinal risk factors and the computation of posterior ORs. The profile regression approach overcomes some of the main limitations of traditional regression methods, like the large number of parameters required when all possible interactions are investigated and problems with inference and interpretation in the presence of collinearity. In the analyzed data, logistic regression analysis (using backward or forward selection algorithms) revealed no statistically significant single or joint effect estimates. In contrast, profile regression identified combinations of covariates that formed subgroups associated with higher or lower risks.

Criticism of MDR focuses mainly on interpretation issues (Manuguerra et al. 2007). Also, when the data are sparse, MDR can be adversely affected, because of the algorithm’s inability to estimate prediction error. This leads to MDR finding solutions with low prediction error even when no such solution exists (Vineis et al. 2008). Low cell counts are less of a problem for profile regression because inference is based on cluster-specific parameters rather than on cell-specific quantities. Results of the classification tree analysis differed from profile regression and were not always consistent with *a priori* expectations. Factors included in a resulting tree, as well as its shape, are sensitive to the choice of criteria used to build and prune a classification tree, and this choice is often not straightforward. In addition, this type of analysis allows for no evaluation of the overall uncertainty.

In previous GEN-AIR analyses, numerous researchers found that NO_2_ and DNA adducts (an intermediate marker of carcinogenesis) were statistically significant predictors of lung cancer in nonsmokers in the same cohort (Peluso et al. 2005; Vineis and Husgafvel-Pursiainen 2005; Vineis et al. 2006, 2007). However, because of small numbers, it was difficult to estimate the joint effect of multiple exposures, and no statistically significant effect of PM_10_ was shown (Vineis et al. 2006; we categorized PM_10_ using tertiles with the lower two tertiles as the reference category, and we included other potential confounders in the model), which was at odds with previous literature (Beeson et al. 1988; Dockery et al. 1993). The profile regression analysis indicated that higher NO_2_ and PM_10_ exposures and residential proximity to roads were more common in the high-risk group. Thus, the profile regression findings for PM_10_ were in agreement with the literature. Moreover, inspecting the genetic profile of the subjects in groups 1 and 2 in Figure 1 gave us an indication, albeit a very weak one, of how the presence of the genetic marker *GSTM1* and the number of DNA adducts may reduce the risk for lung cancer for subjects exposed to air pollution and other environmental attributes. This possible protective effect should be examined in further studies of gene–environment interactions, which would focus on studying the effect of mixtures of air pollutants and their potential interaction with genetic susceptibility, particularly in pathways involved in DNA repair or xenobiotic metabolism.

## Conclusion

We propose that profile regression is a powerful and complementary tool to classical analysis strategy when the focus of the analysis is to characterize the joint effect of multiple risk factors in multifactorial diseases and to identify exposure risk profiles of importance.

## Figures and Tables

**Table 1:** Risk factors included in the profile regression analysis

Risk factor	Categories
Exposure to pollution due to heavy traffic	0, subject does not live on a main road
	1, subject lives on a main road

Exposure to PM_10_	0, < 30 μg/m^3^
	1, 30–40 μg/m^3^
	2, > 40 to 50 μg/m^3^
	3, > 50 μg/m^3^

Exposure to NO_2_	0, < 30 μg/m^3^
	1, 30–40 μg/m^3^
	2, > 40 μg/m^3^

Physical activity at work	0, sedentary occupation
	1, standing occupation
	2, manual work
	3, heavy manual work

Physical activity at leisure	0, 1, 2, with increasing activity from 0 to 2

BMI	0, normal weight
	1, overweight
	2, obese

Deletion polymorphism in *GSTM1*	0, wild type
	1, deletion polymorphism

Polymorphism in the *XRCC1* DNA repair gene	0, wild type
	1, heterozygous or homozygous variant

Information on bulky DNA adducts	0, not detectable
	1, < median
	2, > median

**Table 2:** MDR results (545 subjects)

Model	Prediction accuracy^a^	CVC^b^
ralc	0.50	7/10
ralc, pm10	0.53	6/10
paleis, pawork, no2	0.50	5/10
paleis, pm10, ralc, bmi	0.45	5/10
paleis, ralc, bmi, no2, pawork	0.48	6/10
paleis, ralc, bmi, no2, pawork, x1	0.50	9/10
paleis, ralc, bmi, no2, pawork, gstm1, x1	0.50	6/10
paleis, ralc, bmi, no2, pawork, gstm1, x1, mainroad	0.50	5/10
paleis, ralc, bmi, no2, pawork, gstm1, x1, mainroad, pm10	0.50	10/10

Abbreviations: bmi, BMI: gstm1, *GSTM1* gene; mainroad, residential proximity to a main road; no2, exposure to NO_2_; paleis, physical activity at leisure; pawork, physical activity at work; pm10, exposure to PM_10_; ralc, bulky DNA adducts; x1, *XRCC1* gene.

a An estimate of the predictive ability of the corresponding *p*-factor combination, produced with 10-fold cross-validation.

b Number of times a specific *p*-factor combination was identified as best in the 10 testing sets during the 10-fold cross-validation procedure.

## Appendix

The GEN-AIR collaborators were M. Manuguerra, G. Matullo, F. Veglia, H. Autrup, A.M. Dunning, S. Garte, E. Gormally, C. Malaveille, S. Guarrera, S. Polidoro, F. Saletta, M. Peluso, L. Airoldi, K. Overvad, O. Raaschou-Nielsen, F. Clavel-Chapelon, J. Linseisen, H. Boeing, D. Trichopoulos, A. Kalandidi, D. Palli, V. Krogh, R. Tumino, S. Panico, H.B. Bueno-De-Mesquita, P.H. Peeters, E. Lund, G. Pera, C. Martinez, P. Amiano, A. Barricarte, M.J. Tormo, J.R. Quiros, G. Berglund, L. Janzon, B. Jarvholm, N.E. Day, N.E. Allen, R. Saracci, R. Kaaks, and P. Ferrari.

## References

[b1-ehp-119-84] Albert JH, Chib S (1993). Bayesian analysis of binary and polychotomus response data. J Am Statist Assoc.

[b2-ehp-119-84] Bartsch H (2000). Studies on biomarkers in cancer etiology and prevention: a summary and challenge of 20 years of interdisciplinary research. Mutat Res.

[b3-ehp-119-84] Bastone L, Reilly M, Rader DJ, Foulkes AS (2004). MDR and PRP: a comparison of methods for high-order genotype-phenotype associations. Hum Hered.

[b4-ehp-119-84] BGX (Bayesian Gene eXpression) (2010). Bayesian Integrative Genomics.

[b5-ehp-119-84] Beeson WL, Abbey DE, Knutsen SF (1988). Long term concentrations of ambient air pollutants and incident lung cancer in California adults. Results from the AHSMOG study. Environ Health Perspect.

[b6-ehp-119-84] Berrington de González A, Spencer EA, Bueno-de-Mesquita HB, Roddam A, Stolzenberg-Solomon R, Halkj J (2006). Anthropometry, physical activity, and the risk of pancreatic cancer in the European Prospective Investigation into cancer and nutrition. Cancer Epidemiol Biomarkers Prev.

[b7-ehp-119-84] Bingham S, Riboli E (2004). Diet and cancer—the European Prospective Investigation into cancer and nutrition. Nature Rev Cancer.

[b8-ehp-119-84] Carlsten C, Sagoo GS, Frodsham AM, Burke W, Higgins JP (2008). Glutathione *S*-transferase M1 (*GSTM1*) polymorphisms and lung cancer: a literature-based systematic HuGE review and meta-analysis. Am J Epidemiol.

[b9-ehp-119-84] Cho YM, Ritchie MD, Moore JH, Park JY, Lee KU, Shin HD (2004). Multifactor dimensionality reduction shows a two-locus interaction associated with type 2 diabetes mellitus. Diabetologia.

[b10-ehp-119-84] Cowles MK (1996). Accelerating Monte Carlo Markov chain convergence for cumulative-link generalized linear models. Stat Comp.

[b11-ehp-119-84] Desantis SM, Houseman EA, Coull BA, Stemmer-Rachamimov A, Betensky RA (2008). A penalized latent class model for ordinal data. Biostatistics.

[b12-ehp-119-84] Dockery DW, Pope CA, Xu X, Spengler JD, Ware JH, Fay ME (1993). An association between air pollution and mortality in six U.S. cities. N Engl J Med.

[b13-ehp-119-84] Foulkes AS, De Gruttola V, Hertogs K (2004). Combining genotype groups and recursive partitioning: an application to human immunodeficiency virus type 1 genetics data. Appl Statist.

[b14-ehp-119-84] Gilks W, Richardson S, Spiegelhalter D (1996). Markov Chain Monte Carlo in Practice.

[b15-ehp-119-84] Gupta RC (1985). Enhanced sensitivity of ^32^P-postlabelling analysis of aromatic carcinogen: DNA adducts. Cancer Res.

[b16-ehp-119-84] Hans LW, Ritchie MD, Moore JH (2003). Multifactor dimensionality reduction software for detecting gene–gene and gene–environment interactions. Bioinformatics.

[b17-ehp-119-84] Hoek G, Brunekreef B, Goldbohm S, Fischer P, van den Brandt PA (2002). Association between mortality and indicators of traffic-related air pollution in the Netherlands: a cohort study. Lancet.

[b18-ehp-119-84] Ishwaran H, James L (2001). Gibbs sampling methods for stick-breaking priors. J Am Statist Assoc.

[b19-ehp-119-84] Malats N, Camus-Radon A, Nyberg F, Ahrens W, Constantinescu V, Mukeria A (2000). Lung cancer in nonsmokers and *GSTM1* and *GSTT1* genetic polymorphism. Cancer Epidemiol Biomarkers Prev.

[b20-ehp-119-84] Manuguerra M, Matullo G, Veglia F, Autrup H, Dunning AM, Garte S (2007). Multi-factor dimensionality reduction applied to a large prospective investigation on gene–gene and gene–environment interactions. Carcinogenesis.

[b21-ehp-119-84] Matullo G, Dunning AM, Guarrera S, Baynes C, Polidoro S, Garte S (2006). DNA repair polymorphisms and cancer risk in nonsmokers in a cohort study. Carcinogenesis.

[b22-ehp-119-84] Matullo G, Palli D, Peluso M, Guarrera S, Carturan S, Celentano E (2001). *XRCC1*, *XRCC3*, *XPD* gene polymorphisms, smoking and ^32^P-DNA adducts in a sample of healthy subjects. Carcinogenesis.

[b23-ehp-119-84] MDR (Multifactor Dimensionality Reduction) (2010). Homepage of the Multifactor Dimensionality Reduction Open-Source Software.

[b24-ehp-119-84] Molitor J, Papathomas M, Jerrett M, Richardson S (2010). Bayesian profile regression with an application to the National Survey of Children’s Health. Biostatistics.

[b25-ehp-119-84] Patterson BH, Dayton CM, Graubard BI (2002). Latent class analysis of complex sample survey data: application to dietary data. J Am Statist Assoc.

[b26-ehp-119-84] Peluso M, Airoldi L, Magagnotti C, Fiorini L, Munnia A, Hautefeuille A (2000). White blood cell DNA adducts and fruit and vegetable consumption in bladder cancer. Carcinogenesis.

[b27-ehp-119-84] Peluso M, Munnia A, Hoek G, Krzyzanowski M, Veglia F, Airoldi L (2005). DNA adducts and lung cancer risk: a prospective study. Cancer Res.

[b28-ehp-119-84] R Development Core Team. R (2006). A Language and Environment for Statistical Computing.

[b29-ehp-119-84] Ritchie MD, Hahn LW, Roodi N, Bailey LR, Dupont WD, Parl FF (2001). Multifactor-dimensionality reduction reveals high-order interactions among estrogen-metabolism genes in sporadic breast cancer. Am J Hum Genet.

[b30-ehp-119-84] Seaman SR, Richardson S (2004). Equivalence of prospective and retrospective models in the Bayesian analysis of case-control studies. Biometrika.

[b31-ehp-119-84] Steindorf K, Friedenreich C, Linseisen J, Rohrmann S, Rundle A, Veglia F (2006). Physical activity and lung cancer risk in the European Prospective Investigation into Cancer and Nutrition Cohort. Int J Cancer.

[b32-ehp-119-84] Tibshirani R (1996). Regression shrinkage and selection via the Lasso. J R Stat Soc Series B Stat Methodol.

[b33-ehp-119-84] Veglia F, Loft S, Matullo G, Peluso M, Munnia A, Perera F (2008). DNA adducts and cancer risk in prospective studies: a pooled analysis and a meta-analysis. Carcinogenesis.

[b34-ehp-119-84] Vineis P, Brennan P, Canzian F, Ioannidis JPA, Matullo G, Ritchie M (2008). Expectations and challenges stemming from genome-wide association studies. Mutagenesis.

[b35-ehp-119-84] Vineis P, Hoek G, Kryzanowski M, Vigna-Talianti F, Veglia F, Airoldi F (2006). Air pollution and risk of lung cancer in a prospective study in Europe. Int J Cancer.

[b36-ehp-119-84] Vineis P, Husgafvel-Pursiainen K (2005). Air pollution and cancer: biomarker studies in human populations. Carcinogenesis.

[b37-ehp-119-84] Vineis P, Veglia F, Garte S, Malaveille C, Matullo G, Dunning A (2007). Genetic susceptibility according to three metabolic pathways in cancers of the lung and bladder and in myeloid leukemias in nonsmokers. Ann Oncol.

